# Psychological Wellbeing and Employability of Retrenched Workforce During COVID-19: A Qualitative Study Exploring the Mitigations for Post Pandemic Recovery Phase

**DOI:** 10.3389/fpubh.2022.907797

**Published:** 2022-07-08

**Authors:** Guek-Nee Ke, Dasha Grajfoner, Stephen Carter, Nicole DeLima, Rozainee Khairudin, Wee-Yeap Lau, Khalil Anwar Kamal, Shen Chieng Lee

**Affiliations:** ^1^Department of Psychology, School of Social Sciences, Heriot-Watt University Malaysia, Putrajaya, Malaysia; ^2^Department of Psychology, School of Social Sciences, Heriot-Watt University, Edinburgh, United Kingdom; ^3^Edinburgh Business School, Heriot-Watt University, Edinburgh, United Kingdom; ^4^Centre for Research in Psychology and Human Well-Being, Faculty of Social Sciences and Humanities, National University of Malaysia, Bangi, Malaysia; ^5^Department of Applied Statistics, Faculty of Economics and Administration, University of Malaya, Kuala Lumpur, Malaysia; ^6^Malaysian Institute of Economic Research (MIER), Kuala Lumpur, Malaysia

**Keywords:** retrenchment, COVID-19, psychological wellbeing, job loss, mental health, post-pandemic recovery, latent-deprivation model

## Abstract

The ongoing COVID-19 pandemic has triggered several employment-related issues, followed by different response strategies. With retrenchment measures being a common response strategy during economic downturns, many individuals have been faced with unemployment. This study aimed to explore the effect of the COVID-19 pandemic related retrenchment on the psychological wellbeing of the Malaysian workforce, using a qualitative research approach and based on the Latent-deprivation Model. A purposive sample of 30 retrenched participants was interviewed via telephone during Malaysia's Movement Control Order (MCO). Thematic analysis was subsequently conducted to identify key themes in the data set. Six themes emerged from the thematic analysis: (1) Retrenchment leading to the deterioration of psychological wellbeing; (2) Unemployment, financial strain and economic uncertainty; (3) Emotions related to the COVID-19 virus; (4) Coping with lifestyle change; (5) Recommendations to improve the psychological wellbeing and mental health of the retrenched workforce, and (6) Career and financial support at the recovery phase. The present study provides valuable insight into the wellbeing of retrenched workforce who are forced to cope with both unemployment and a global pandemic, and workforce perspectives regarding types of support needed during the recovery phase.

## Introduction

Retrenchment is one of many serious employment issues facing globally during the COVID-19 pandemic. While lockdown and quarantine measures are essential for preventing the spread of the infection, the contagion is still growing, albeit slower than in 2021, and led to the abrupt halt in the growth of the global economy, resulting in economic downturn (1, 2). The economic affected nations include the G7 nations (United States of America, China, Japan, Germany, Britain, France, and Italy) which account for 60% of world supply and demand, 65% of world manufacturing, and 41% of world manufacturing export as well as a substantial part of global value chains. Such disruption has affected the Gross Domestics Product (GDP) deficit, estimated at different degrees of severity from −2 to −10% (1). As a result, businesses of all sizes and in all sectors have utilized a variety of strategies to survive through the ensuing economic crisis, including downsizing their workforce (3, 4). Retrenchment is a common response strategy used to reduce costs to stabilize declining performance, reduce complexity by focusing on existing activities, and ultimately, restore profitability (5, 6). Under the current COVID-19 pandemic, massive retrenchment measures have been conducted across the world, leaving many individuals unemployed (3). In Malaysia, the unemployment rate had shot up from 3.9 to 5% in April 2020, peaking at 5.3% in May 2020 (7). Among retrenched Malaysians, a large proportion reported experiencing severe depression, anxiety, and stress (8, 9). Recent reports have predicted deterioration in mental health as a secondary crisis following health worries and economic fallout of the COVID-19 pandemic (10). To the authors' knowledge there is currently no qualitative data on the effect of unemployment during the COVID-19 pandemic on psychological wellbeing of the Malaysian workforce. Therefore, this study aims to fill this gap by using semi-structured interviews and thematic analysis. In addition, this study also aims to explore suggestions regarding types of support needed to improve the psychological wellbeing through the lens of the retrenched workforce.

The research approaches the topic from the Latent-deprivation Model (11) point of view. The Latent deprivation model illustrates that employment is the core provider of five sub-constructs of experience, namely time structure, social contact, collective purpose, status, and activity. These experiences strongly link to individual mental health (11–13). Numerous scholars have supported Jahoda's studies in that the lack of latent benefits of work associated with unemployment, leads to job insecurity, mental and psychological distress (14–19). Hence, this study explores the Latent-deprivation Model's assumption that the psychological health experience by retrenched workforce can be explained by a deprivation of latent benefits of employment. This paper reviews the psychological wellbeing and employability of the retrenched workforce during the COVID-19 pandemic, mitigations for post pandemic recovery approach, as well as the role of psychological fulfillment in Jahoda's latent-deprivation model.

## Literature Review

Over the past decades, there exists a vast literature on unemployment, psychological distress, and their co-occurrence. These studies examine the mechanisms of unemployment which leads to psychological distress and supported by various models of employment. The literature, therefore, looks at the causal relation between the world economic downturn following the COVID-19 pandemic and its effects on psychological wellbeing.

### The Effect of Unemployment on Psychological Wellbeing

According to the Latent-deprivation Model (11), work provides both manifest functions (i.e., financial income) and latent functions (i.e., a daily routine, socialization, purpose, social status, and regular activity). The loss of latent functions, in particular, negatively affects psychological wellbeing (15, 16). On a similar line of thought, numerous studies (20, 21), suggested that working has the potential to fulfill three essential human needs; firstly, being the ability of afford important resources for continued survival, such as food and shelter. Secondly, being the opportunity to experience self-determination (i.e., competence, autonomy, and relatedness) which can be fulfilled through work (22). Thirdly, the ability to access social support and make real-world connections. As work means a great deal to society, retrenchment globally has further added to the existential fear and worries already experienced due to the fear of premature death due to COVID-19 (23). Likewise, Warr (24, 25) highlighted that work leads to twelve benefits, including opportunity for control, opportunity for skill use, externally generated goals, variety, environmental clarity, availability of money, physical security, interpersonal contact, valued social position, supportive supervision, good career prospects, and fair treatment. Hence, these studies highlight that positive outcomes brought by employment are invaluable.

To further understand this effect, unemployment has been found to be associated with poorer psychological wellbeing; with subsequent re-employment improving mental health (18, 26–31). Unemployed individuals have reported experiencing an increase in psychological distress, depression, anxiety, and psychosomatic symptoms, as well as loss of confidence and self-esteem following unemployment (18, 29, 31, 32). Past studies show that unemployment following a recession negatively affected psychological wellbeing (33, 34), with a global trend of job loss being associated with an increase in suicides (35–38).

Unemployed individuals reported that work had provided purpose, as well as maintained their personal identity and self-worth (33). Furthermore, unemployed individuals also described feelings of helplessness and isolation, which reinforced their loss of self-esteem and produced feelings of worthlessness which caused them to lose a sense of belonging within the workforce (17, 33, 34). From this evidence, it can be inferred that without work, individuals would be deprived of both material and psychological needs.

### The Effect of Financial Strain and Economic Uncertainty on Psychological Wellbeing

Having established unemployment as one key issue, it is possible to consider others of equal challenge; financial strains, and economic uncertainty. The World Bank (39) has recently predicted that the COVID-19 pandemic has triggered one of the deepest recessions in decades and will leave lasting effects on the global economy, despite the optimism that the vaccination programme may bring in those economies lucky enough to afford the rollout, for example the USA and the UK, or those who managed the pandemic efficiently, for example Australia and New Zealand. Based on previous studies, investigating the Great Recession in the late-2000's [e.g., (27, 40–44)], it is well-documented that economic recession led to organizational downsizing, mass layoffs and higher unemployment rates, inciting a rise in psychological distress and common mental disorders, substance disorders, as well as the increased risk of suicide. A rise in depression and anxiety disorders are often reported after economic breakdowns caused by disasters (45).

The economic uncertainty due to the COVID-19 pandemic has led to organizational downsizing and mass layoffs globally (3, 4). The unpredictability and rapid changes in the labor market have led to panic over job uncertainty, and uncertainty of the future in general (46). Intolerance of uncertainty has been found to be associated with worry, depression and anxiety disorders, where the greater the perceived uncertainty stress, the higher the prevalence of mental disorders (14, 46–49). In addition, experiencing job uncertainty will inevitably affect one's identity, self-efficacy beliefs, confidence, and social support system (46).

As stated previously, work acts as a source of income (20, 21). A sudden loss of income due to retrenchment was found to negatively impact psychological wellbeing, with financial strain being the mediating factor (17, 50–54). This is in line with the Latent-deprivation Model (11), and supported by Hobfoll's (55) Model of Conservation of Resources, whereby loss of economic resource leads to negative psychological outcomes (56). Not only does sudden financial strain affect the ability to afford food and shelter for survival, but it also reduces freedom in making decisions, which negates the human desire for agency and self-directedness (15, 51, 57). Such financial difficulties may also prevent individuals from accessing psychological support (58). In fact, the first cases of suicide due to financial hardships related to the COVID-19 pandemic have already been reported in Bangladesh and Pakistan (59, 60).

The effects of financial strain on psychological wellbeing are prolonged if individuals experience difficulties in finding a new job (51). Furthermore, unsuccessful job searching and the lack of control over the job search led to feelings of despair and frustration (51, 61). As a result, fear of not securing a new job has been found to negatively affect physical health (34). From this, it can be concluded that financial strain and economic uncertainty post a great impact on individual psychological wellbeing.

The evidence highlights that the COVID-19 pandemic has triggered the economic downturn, and for businesses sustainability, many organizations across the globe employ retrenchment as a core strategy that leads to unemployment. Hence, this phenomenon not only deprived the latent manifest and functions of employment, but also the psychological wellbeing. For this reason, the authors propose a conceptual model to explore the experience and recommendation of the retrenched workforce to mitigate and improve overall landscape.

### The Conceptual Model

Jahoda (11) argued that paid work provides both manifest (associated with income) and latent (associated with fulling psychological needs) benefits. While working, an individual profits from the five latent benefits of time structure; social contact, common goals, status, and activity. Hence, it is evidenced that deprivation of employment leads to deprivation in both manifest and latent benefits, but it is the loss of the latent benefits that have an adverse impact on psychological wellbeing.

Comprehensive research studies on the Latent-Deprivation Model can be traced over the past two decades 2000–2009 and 2010–2019. The early part of the 2000–2009 decade saw the significant predicted breakthrough of unemployment with wellbeing (50, 62), psychological distress (63), mental health (64, 65). In the following decade 2010–2019, research on Latent-Deprivation Model expanded its scope looking into diversity and cultural difference (66) and longitudinal study (17). Results further evidenced an association between deprivation of unemployment and psychological health. Continuous research was conducted (starting in the 21st century, 2020–2022), covering both the Latent-Deprivation Model and psychological wellbeing in different contexts (16).

Research to date have largely tested the latent benefits on psychological wellbeing using quantitative approaches such as the cross-sectional design with survey and questionnaires such as General Health Questionnaire (50, 62, 66), Profile of Mood Stage and Global Self Worth Scale (63), Depression Scale (64). Nonetheless, these self-administered studies were lacking in-depth exploration on psychological health. Similarly, real time strategies to mitigate the situation were non-existant.

This present study extended the methodology using a qualitative approach interview with interpretative phenomenological analysis. An alternative perspective is given by this study, we argue that psychological fulfillment can be a potential new latent variable for the Latent-Deprivation Model. Furthermore, this study extends possible mitigations in a real time context and at the post pandemic recovery phase.

**Figure 1:**
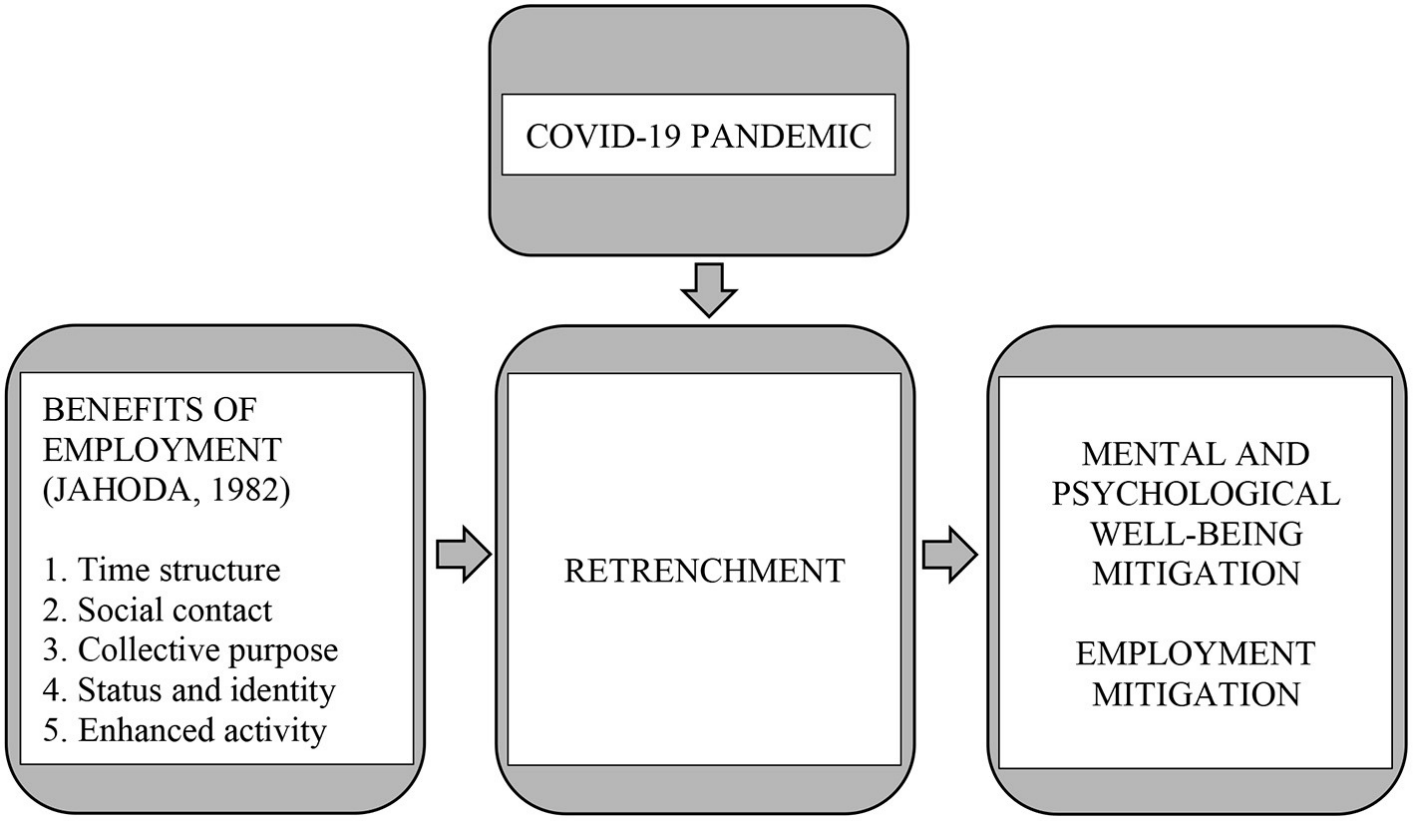
Conceptual model.

To summarize, this study provides a novel context under the unprecedented COVID-19 pandemic. The workforce's experience on retrenchment, which leads to unemployment during COVID-19, may provide a new insight to the perspective when interpreting the Latent-Deprivation Model. Having looked at the development of the Latent-Deprivation Model and relevant literature, the following conceptual model, using a derivation of the Latent-Deprivation Model, was developed (Figure 1). This present study explores the effect of retrenchment on psychological wellbeing.

## Method

### Study Design

A qualitative approach was used in this study to explore the effect of retrenchment due to COVID-19 pandemic on psychological wellbeing (67). To encourage participants to share their experiences, semi-structured interviews were conducted (67). Interpretative phenomenological analysis (IPA) was performed to explore participants' perceptions of their experience being retrenched (68). Data from interviews were transcribed, coded, and analyzed for patterns, which eventually lead to the identification of themes. The coding process and the forming of themes were completed using NVivo software version 12.

### Study Sample

In IPA research, smaller concentrated samples are recommended with an average participant size between one and twelve (69). This study aimed to draw upon a sample which enabled the generation of sufficient data for an in-depth investigation, hence 30 participants were interviewed (68). A purposeful sampling strategy was applied in recruiting homogeneous participants who would be able to provide insight to this study (70). All participants (Table 1) fulfilled the inclusion criteria, which included being a Malaysian citizen, age 18 and above, and had experienced retrenchment and loss of job as a result of the COVID-19 pandemic in Malaysia. An advertisement poster inviting qualified participants to register for the study, was distributed at government agencies, non-profit organizations (NGOs), and social media.

**Table 1:** Demographics of the participants.

		**Frequency, *n***	**Percentage (%)**	***SD***
Gender				–
	Male	15	50.0	
	Female	15	50.0	
Age				5.75
	21–30	24	80.0	
	31–40	4	13.3	
	41–50	2	6.7	
Ethnicity				–
	Malay	15	50.0	
	Chinese	8	26.7	
	Indian	7	23.3	

Participants consisted of 15 males aged 23–43 years (Median = 27) and 15 females aged 21–42 years (Median = 25). Of the 30 participants, 50% were of Malay descent (*N* = 15), 26.7% were of Chinese descent (*N* = 8), and 23.3% were of Indian descent (*N* = 7). The proportion of participants in this study corresponded to the population of Malaysia, which comprises of 67.4% Bumiputera, 24.6% Chinese, and 7.3% Indian (71). The participants were from service industries such as financial, consultancy, and healthcare, the majority of which are private organizations 66.7% (*N* = 20) and small-medium enterprises 20% (*N* = 6).

### Data Collection

Participants who registered their interest to take part in this study were contacted by telephone. To ensure eligibility, participants were asked if they had been retrenched as a result of COVID-19 pandemic. Interviews were conducted via telephone, as at the time of the study, Malaysia was under the Movement Control Order (MCO)—a preventative measure by the federal government of Malaysia to control the spread of COVID-19. Mass movements and social gatherings were prohibited at the time.

Prior to the interview, an information sheet explaining the aim of the study, a participant consent form, and a participant consent form for audio recording were sent to participants via email. Interviews took place between 19th June and 27th July 2020. Each interview lasted between 40 and 60 min. All interviews were audio recorded with permission.

The interview questions were designed based on the Latent-deprivation Model's assumptions; the psychological health experience of the retrenched workforce can be explained by a deprivation of latent benefits of employment.

1. What is your experience with government measures related to MCO?
2. What challenges have you experienced due to pandemic crisis and MCO?
3. What kind of psychological support will be most useful?
4. What kind of training or personal development would be beneficial at this particular time?
5. Do you have any hopeful suggestions that would improve your psychological wellbeing and the quality of life?

## Results and Discussion

**Figure 2:**
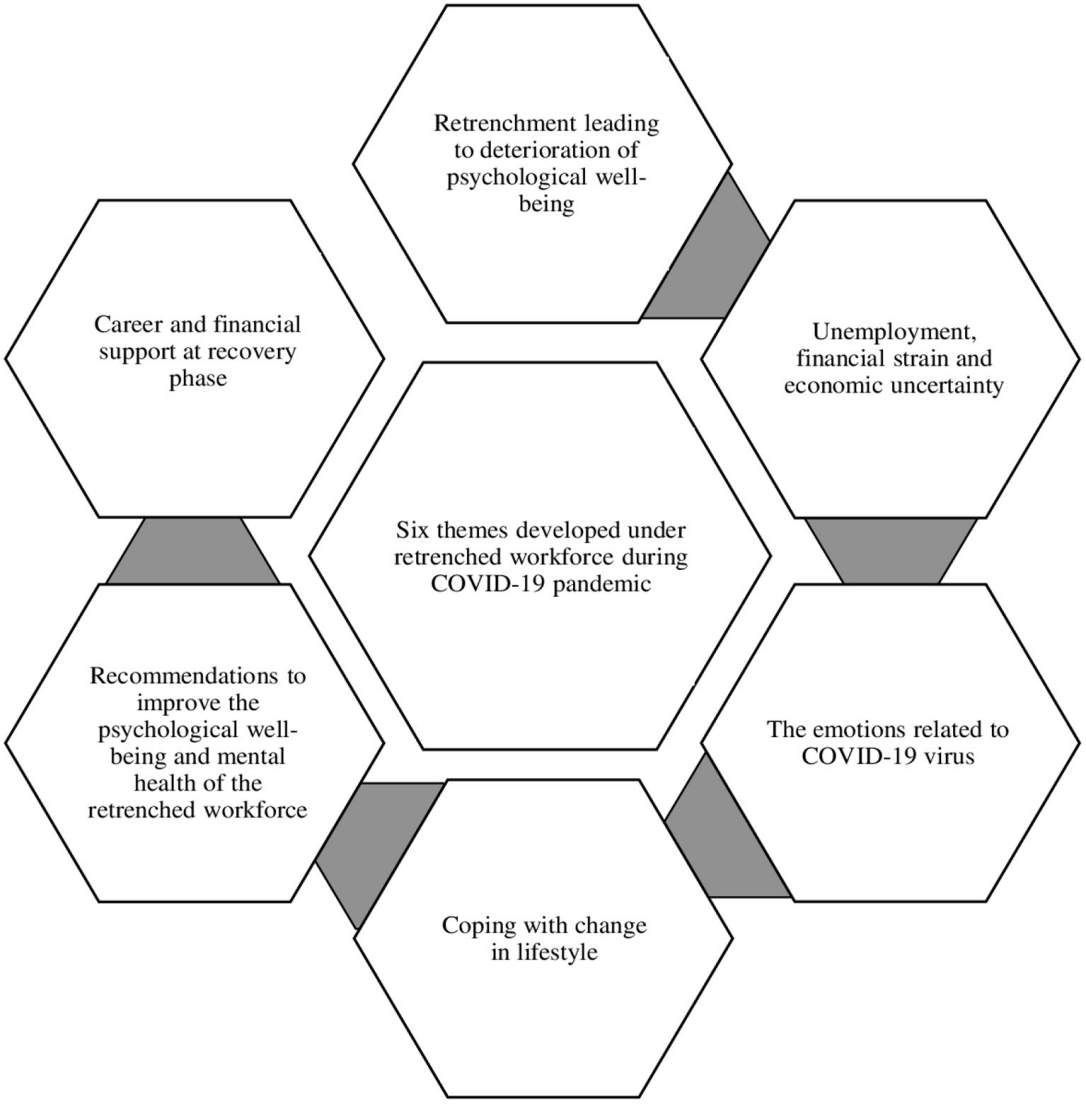
Diagram illustrating inter-connected themes related to retrenched workforce.

The thematic analysis revealed six different themes (Figure 2), (1) Retrenchment leading to deterioration of psychological wellbeing; (2) Unemployment, financial strain and economic uncertainty; (3) The emotions related to COVID-19 virus; (4) Coping with change in lifestyle; (5) Recommendations to improve the psychological wellbeing and mental health of the retrenched workforce; and (6) Career and financial support at recovery phase.

### Theme 1: Retrenchment Leading to Deterioration of Psychological Wellbeing

Consistent across all interviews, and in line with the Latent-deprivation Model, retrenchment leading to unemployment was found to be one of the main stressors affecting psychological wellbeing following retrenchment. Previous studies have also found that unemployment negatively impacts mental health and wellbeing (18, 26–31, 33, 34).

Almost all participants reported that losing their job had a negative effect on their mental health. Participants reported feeling depressed, shameful, frustrated, and helpless due to the loss of their jobs. Most participants found it difficult to cope with their sudden unemployment.

*(Participant 22) ‘After the retrenchment, it was also very difficult to cope with—because it was a sudden shock that you just lost your security and your work and everything. So, that was definitely a big toll to take. […] I spent a lot of time in my room being alone because I didn't want to face the reality of it'*.

They further described a feeling of defeat from applying for jobs and facing countless rejections. This had caused feelings of helplessness and hopelessness.

*(Participants 10) ‘It has taken a bit of a mental toll in the sense because having being retrenched, having to go through the whole stress of reapplying for jobs and getting rejections, and trying to figure out how to get hired again. […] For me, I've been applying [for] jobs steadily since May, since I knew that I was getting laid off, and some days do get a little bit difficult—in terms when we wake up, it kind of feels a bit hopeless'*.

However, some participants reported coping well with their retrenchment. They did not attribute retrenchment to themselves. In contrast, they reasoned that their job loss was due to external circumstances.

*(Participant 23) ‘I know the reason why my company had to shut down. I know why everyone lost their job. […] The effect of this pandemic was really bad and everyone is affected. Whichever industry you're in, it will be affected—either it is completely shut down or half-shut. For me, I cannot run from the reality. Because my company […] does not have any chance of surviving through this'*.

### Theme 2: Unemployment, Financial Strain, and Economic Uncertainty

All participants described that their main worry was related to their socioeconomic status. The sudden loss of income due to retrenchment left them unprepared, and unable to support themselves or contribute to household income. Similar findings were presented by past studies, which further suggested that it would be inappropriate to disregard the role of money in present-day capitalist culture. Loss of income causes financial strain, inevitably affecting standards of living, and ultimately psychological wellbeing (17, 33). The results supported the Latent-deprivation Model (11), where a loss of economic resource led to negative psychological outcomes (16, 56).

Participants financial worries included paying bills, paying rent, buying groceries, and paying loans. Many participants were forced to live off their savings.

*(Participant 23) ‘[…] financially, I am not prepared because it all happened suddenly. […] MCO started after Chinese New Year, around the 27th or 28th. Then about two weeks after, we were locked down. I couldn't do anything. […] And then after two months, the company shut down. […] Financially, there was none. My savings had reduced during the MCO'*.

Participants also reported, due to deprivation from financial income, their plans to pursue further studies, get married, purchase of house and car were on hold for the time being. The financial strain experienced had caused anxiety, as well as feelings of helplessness and desperation. Financial strain persisted as they were unsuccessful in securing a new job (17, 19).

*(Participant 24) ‘[…] getting married, propose, buy a new house, buy a new car—all those things were in this year's plan. […] All that will be put on hold indefinitely. […] It is obvious that companies right now are retrenching and not hiring. [In] a moment of helplessness, […] I didn't know what to do. […] I was really desperate. I was just looking for any job'*.

Due to financial strain, they could no longer access mental health services.

*(Participant 3) ‘It would be nice to go back to therapy but [at] the end of the day, if you want to go [to] therapy, you need to have money'*.

Participants felt that it was important for them to secure their basic needs (i.e., job security and financial stability) before addressing psychological needs. Any support financially would improve their mental health.

*(Participant 30) ‘You need to fulfill your most important needs—the first level. Once you have fulfilled that need, then only comes the next level. Like myself, the most important is to find a job. […] I mean we have to look at the hierarchy. […] mental health is secondary'*.

Another factor found negatively affecting psychological wellbeing in this study, was economic uncertainty. The majority of participants described feeling anxious about economic uncertainty, as well as uncertainty of the future, in general. It prompted participants to display anxiety-related behaviors, such as nail biting, overthinking, and trouble sleeping, as well as panic attacks.

*(Participant 14) ‘For me, it's just the anxiety of not knowing what will happen next and whether or not I'll be able to sustain my current lifestyle. Just the anxiety and instability of financial income at the moment and in the future, and not knowing when will […] industries open up again. Because right now everything is closed down and no one is hiring'*.

Several of the participants reported feeling anxious and overwhelmed when watching daily press briefings, or when receiving updates on social media. They found it difficult to cope with the stress of the uncertainty of the ongoing situation.

*(Participant 10) ‘More than anything—more [than] health or the actual risk of being infected—it's more so the fear that affects people and takes over the psyche. […] when you see all these news in Malaysia, news reports and daily press briefings, and all the news coming from the world and social media, things like that—it does play a part'*.

With the rapid outbreak of the COVID-19 pandemic, it brought about a rise in economic uncertainty shock; even larger than that of the Great Recession (72). As accurately anticipated by Godinic et al. (46), participants reported feelings of anxiety due to economic uncertainty, as well as uncertainty of future in general. This finding supported past literature on intolerance of uncertainty, whereby intolerance of uncertainty is highly related to worry and stress-induced anxiety disorders (47, 48).

### Theme 3: The Emotions Related to COVID-19 Virus

Emotions related to COVID-19 had also emerged as a challenge. Most participants in the study described feeling worried about being infected themselves, or that a family member would be infected. In some cases, they were expressing the desire to avoid contact with family members who were frontliners, working in a customer-facing industry, living abroad, advanced in age, or have a chronic health issue. These findings are in line with studies on previous outbreaks (73, 74).

Some participants expressed that they were not worried about being infected as they followed the Standard Operating Procedure (SOP) provided by the Government, or they believed that they were not at exposed to the virus.

One participant described feeling traumatized when they developed symptoms of COVID-19 after attending an event just before MCO.

*(Participant 18) ‘So, I had a […] slightly traumatic experience because I went for an event—I had a work event just before the MCO and right after I started having symptoms. So, I had breathlessness, I had chest pains, I had flu-like symptoms. So, I actually started quarantining myself two days before the MCO started. […] In my case, I thought I was infected and I had symptoms for a month—more than a month. […] It got even worse. And so, I was emotionally affected by that. […] I was really afraid'*.

Another participant described feeling paranoid about being infected with COVID-19 after falling ill with a normal fever.

*(Participant 20) ‘So, at first, when it happened, I started to become a little paranoid, and then I started getting all these types of fever and sore throat. So, that's when I felt like I might have it. After medication and calming down myself, it was just a normal fever. So, once it all went away, I'm actually okay'*.

### Theme 4: Coping With Change in Lifestyle

While quarantine measures are necessary to control the outbreak, it has been found to negatively affect psychological wellbeing [e.g., (75, 76)]. Coping with changes in lifestyle due to quarantine had emerged as a factor negatively affecting psychological wellbeing. Confined to their own homes, participants described feeling bored and trapped. They also reported feeling isolated and disconnected from others, as their social and physical contact had reduced which is in line with latent deprivation of social context (11). Like many other countries, due to lockdown measures, participants were prohibited from leaving their homes without a valid reason. Participants described feeling bored and restless, as the daily routine during MCO quickly became repetitive. They also described feeling trapped and a reduced agency over their actions.

*(Participant 24) ‘It was very repetitive. It's very mundane. I wake up, I do the same thing. […] I think at a certain point I felt trapped as well. I was just in my room the whole time. Every day I'm just in these four walls and I'm looking at […] my MacBook, […] and if it's not that screen, I'm looking at my phone, and if I'm not looking on my phone, it's just another screen […]'*.

*(Participant 6) ‘It was challenging. It was a surprising discovery of character that I have experienced. I have always considered myself as someone who prefers to stay in rather than go out, but I found that when the option is being taken away from me, it left me feeling one thing. I felt very controlled. […] I felt like it was a feeling of loss of self-control'*.

Due to lockdown measures, participants reported a reduction in social activities. Without real-world connections, participants also described feeling isolated and disconnected from others. They reported an increase in online communication, though this did not replace the need for physical connection.

*(Participant 24) ‘I feel like I'm really disconnected [from] the world. As much as I'm connected to the internet […], it just feels really distant. I don't feel the connection. […] it feels really different because there's no physical connection'*.

This was particularly difficult for those who were separated from family, which was also reported by Hawryluck et al. (75).

### Theme 5: Recommendations to Improve the Psychological Wellbeing and Mental Health of the Retrenched Workforce

As many were unable to access mental health services, participants suggested more affordable therapy session or subsidies for mental health services to be provided. They suggested organizing training or workshops on mental health, specifically workshops on understanding emotions, on how to cope with change and low moods, as well as on how to take care of their mental health and family members. Having established the importance of psychological intervention, it is possible to consider such intervention underpinning positive psychology theories and approaches which also has proven to be beneficial for individual to regain life purpose (77, 78). According to Maier and Seligman (79), learned helplessness leads to the belief that nothing one does matters. They theorized that subjective helplessness was cognitive and that it was learned. This concept explained the retrenched workforce's situation under the COVID-19 pandemic. Pioneers in Positive Psychology (81, 82) stated that positive psychology intervention contributes to psychological fulfillment.

The majority of participants also shared that they did not have any well-defined goals and expressed their interest in career counseling/coaching for guidance, moving forward, in their professional lives. They felt they needed advice on topics such as setting career goals, applying for jobs, choosing a career path, switching careers, as well as starting and growing a business.

*(Participant 20) ‘So, maybe they should do a little workshop for people because [workshops are not only] important for those who actually have the mental health problem, but for family and friends, who don't know that their loved ones are having these mental issues, maybe [they]can go to this workshop, and learn about these things […] to understand their friends and family better—those who are having the mental health [issue]—instead of shutting them down'*.

Participants also suggested organizing more outreach and re-integration programs for the retrenched workforce. They shared their own experiences with overcoming mental health issues and the difficulty they faced re-entering their community.

*(Participant 28) ‘[…] it's very hard for me to plan out my next step—to take control of my life again. I feel like if there were NGOs or Government-related program that can help with that, I feel like that would be something that Malaysians could benefit from. […] I feel like if there were programs or service available to provide that kick start to get back on track that would be beneficial'*.

In addition, participants suggested improving awareness of mental health issues, reducing the stigma against therapy via social media and other forms of media.

*(Participant 26) ‘Maybe advertise in social media, or television. Maybe that can remove all the negative stigma [among] Malaysians. […] Because [Malaysians] assume everyone going to hospital—going for mental checks—is crazy. […] The first thing to do is telling people that mental health is just a disease—not that someone is crazy. Fixing the stigma will be the first thing to do'*.

Besides that, a suggestion was for forming support groups for retrenched individuals, in order to give them the opportunity to meet other individuals who were going through a similar situation. It would also help promote a sense of togetherness, and combat feelings of loneliness and helplessness.

*(Participant 1) ‘I guess a peer-to-peer support will be quite useful. Some sort of group session where people […] could share what they are doing to mitigate the crisis, I think that would be fantastic and also, I think that would be inspirational to some people if they could talk about their success stories during MCO—they got retrenched, they got fired, but these are the things that they did to sort of mitigate the risk to themselves and how they could improve their lives'*.

*(Participant 22) ‘Yes, it would be very helpful. Because I think there would be a sense of togetherness— something to feel less alone and helpless. […] I think it will help people just cope during the whole situation'*.

In Jahoda's model, employment fulfills the latent functions' which result in good mental health, and vice-versa. To put it another way, improvement of latent functions of unemployment can be achieved by scrutinizing the psychological support system and intervention. Such processes would help to improve the retrenched workforce's mental and psychological wellbeing by focusing on psychological gains following unemployment. Moreover, it offers insights on the psychological meaning of employment, which is considered an important source for fulfillment of psychological needs (17, 64).

### Theme 6: Career and Financial Support at Recovery Phase

This finding supported the Latent-deprivation Model where income is one of the manifests for latent function (14, 15, 19). To regain the latent benefits of employment, participants suggested four main ideas—first, financial aid provided by local and federal government; second, minimum wages; third, job searching support and lastly, skill-based training.

As participants had lost their source of income, many relied on the financial aid provided by the Government. While many shared positive experiences with the financial aid, a subset of participants suggested that more financial aid should be provided, as the aid already awarded was not sufficient to cover the expenses of some individuals and families. Besides that, participants suggested that increasing the minimum wage or basic salaries could potentially help reduce the financial burden that most Malaysians are currently facing.

*(Participant 10) ‘I think what is very important for countries like Malaysia is to increase things like minimum wage, to increase base salaries. I do believe that in a COVID-19 situation or any pandemic situation if you earn [enough] money [through] work, then of course it is going to be less of a financial burden to you if you are retrenched, or if you get your salary reduced or cut [during] this period. At least, you still have enough income that [retrenchment or pay cuts are] not going affect too much'*.

As most participants, at the time of interviews, were searching for jobs unsuccessfully, they felt that they could benefit from more support in job searching.

*(Participant 10) ‘For me, what would help is support when it comes to, say for example, helping to find a job or a job placement, and things like that. So, that would ease a lot of the burden I'm going through'*.

Participants also proposed that more skill-based training or workshops should be organized to allow Malaysians to gain new skills or retrain for new jobs.

*(Participant 10) ‘[…] retrain retrenched workers whose industries were hugely affected […]. Retraining is a big initiative that I think the Government, and also private sectors, should definitely jump onto and assist'*.

**Figure 3:**
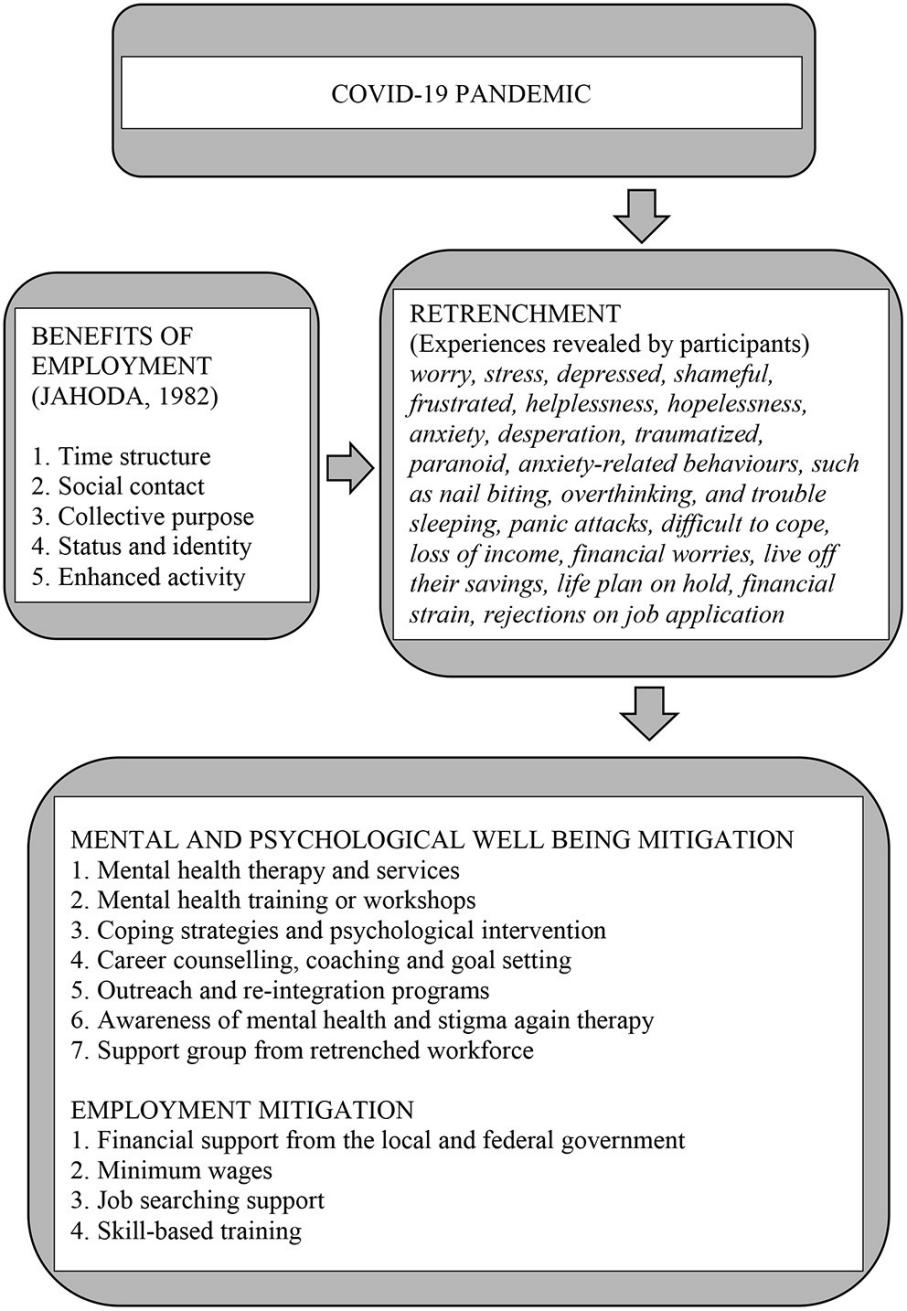
The proposed framework.

From the established themes, in line with the Latent-deprivation Model it can be concluded that employment does provide latent and psychological benefits. The findings supported the conceptual framework (Figure 1), for example the retrenched workforce experienced various adverse effects, both psychological and unemployment, brought on by retrenchment during the COVID-19 pandemic. Hence, in the light of the study results, the conceptual framework was revised to present a proposed framework which highlights the adverse effect of psychological wellbeing and unemployability, and, at the same time, shed light on mitigation at the recovery phase and future resilience (Figure 3).

## Implications for Theory and Practice

### Implications for Theory

The focus of this study was to gain a deeper understanding and greater insight into the participants' experiences of retrenchment, adding richness to our understanding of Jahoda's Latent-deprivation Model and about how workplace retrenchment can deteriorate workforce's psychological wellbeing (26, 27, 33, 34). The study findings further support the Latent-deprivation Model (11) whereby psychological needs are unable to be satisfied without work.

The findings in this study suggest that loss of manifest function negatively affects psychological wellbeing just as much, if not more than, the loss of latent functions. In this study, financial strain was found to be a prominent stressor following retrenchment, supporting findings from previous studies (50–54). This finding also in line with Fryer (57), who proposed loss of income most negatively affected psychological wellbeing.

**Figure 4:**
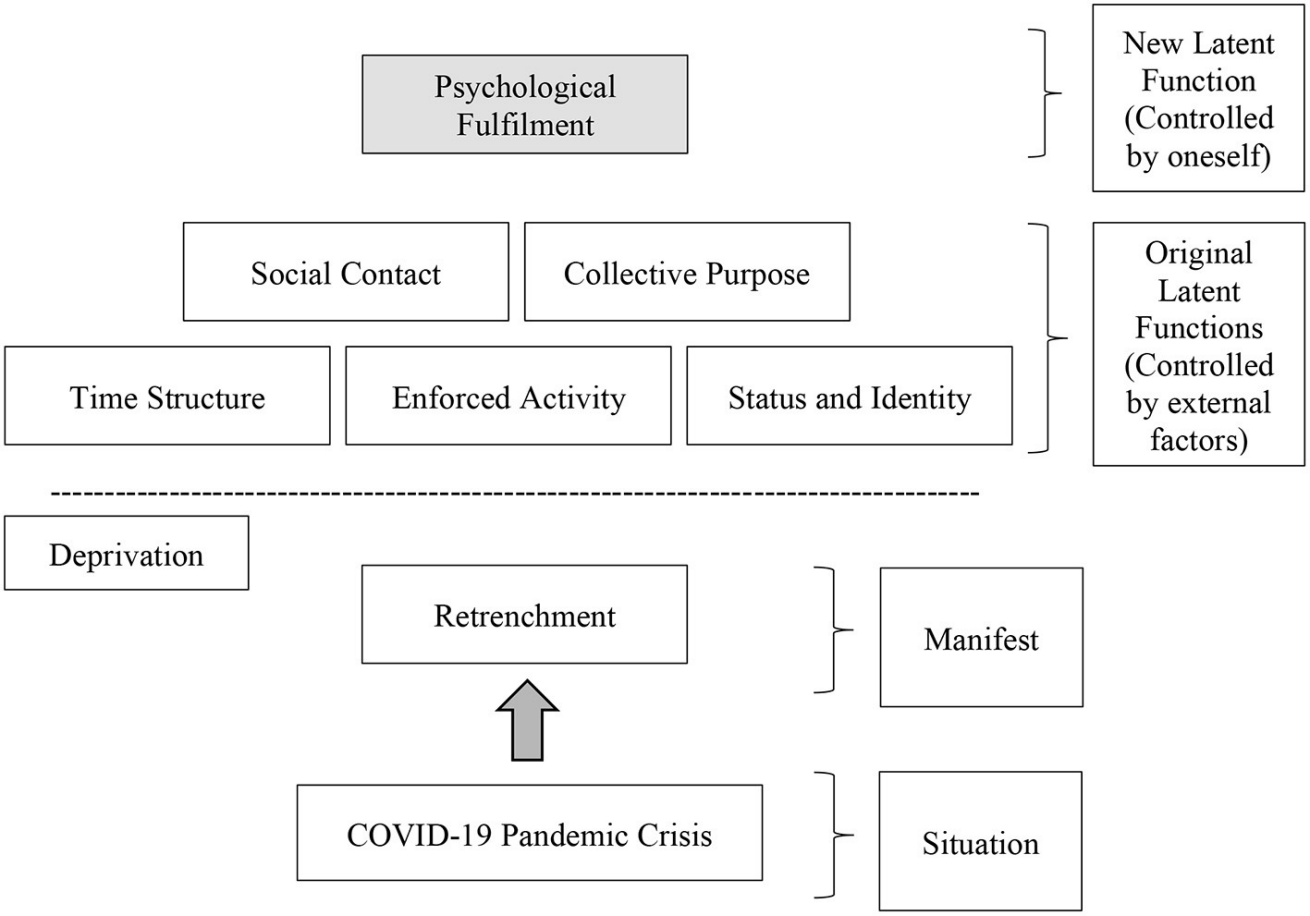
Proposed latent-deprivation model of retrenchment under COVID-19 pandemic crisis.

The evidence suggested that psychological fulfillment could be embedded as part of the Latent-deprivation Model, not as an external casual factor, although due to the small sample and its' geographic location, this is a tentative suggestion only. Hence, Figure 4 shows the current Latent-deprivation Model but adds the psychological fulfillment element.

Based on Figure 4, the situational factor (COVID-19 pandemic crisis) triggered the “manifest of action,” i.e., workplace retrenchment and so subsequently caused unemployment. For this reason, the retrenched workforce is deprived of the latent benefits of employment; which includes psychological fulfillment as evident by this study and other scholars (18, 26, 27, 29–31, 33, 34, 80). From this, it can be rationally inferred that the inclusion of psychological fulfillment as a new latent, and the only latent, that is learnable and controllable by an individual, is reasonable (81, 82).

### Implications for Practice

At least four implications for practice can be suggested. First, these findings warrant the attention from decision-makers, especially the government and employers, to meet the needs of the retrenched individuals with actions aimed to deal with issues of unemployment and psychological wellbeing nationally and globally; ultimately contributing to the betterment of public health. This includes the inclusion of mental health coverage in insurance programs for retrenched employees as a mandatory employment policy by the government.

Second, mental health practitioners are encouraged to apply a purposeful designed “Positive Emotion-Resilience-Self Efficacy (PERCE) Model” in their professional practices as a post-pandemic recovery approach (9). Research on positive emotion evidenced the association to improve reactions to aversive stimuli and promote resilience (83). Moreover, scholars supported that enhancing resilience is associated with better mental wellbeing among the workforce (84, 85). A subsequent study proved employee resilience was positively linked to mental health and psychological wellbeing (86). In addition to positive emotion and resilience, individual coping self-efficacy specifically addresses one's confidence in the ability to cope effectively when facing challenges (87). Hence, mental health professionals, including psychologists, should have an interest in supporting the retrenched workforce to be effective, whilst also paying attention to their psychological wellbeing, especially during times of the COVID-19 pandemic. For examples, coaching psychologists, occupational psychologists, and counseling psychologists should be encouraged to apply the PERCE Model to overcome the psychological distress and in setting new life direction for the retrenched workforce in the post-pandemic recovery phase (9), which could be embedded in coaching psychology techniques and approaches (88).

Third, the corporate responsibility of human resource management is to establish a dedicated mental-health support system for the retrenched workforce under the Employee Assistance Programme (EAP) after the retrenchment process, ideally for 6 months. Hence, the purposeful and extended EAPs for the retrenched workforce would be designed to facilitate and promote retrenched workforce wellbeing. A compassionate discharge of the workforce should look not only at financial compensation but also psychological readiness. Appropriate programmes and services to this end, should include professional assessment, coaching, mentoring, and counseling directed at retrenchment or psychological health (89, 90). Hence, this mitigation is important for the workforce's psychological readiness in the recovery phase.

Lastly, through the lens of human resource management, it is of the utmost importance to the cultivation of a positive organization, to form a resilient organizational ecosystem for talent sustainability (91). Positive organizational culture such as gratitude, kindness and flow has been evidenced to be effective by organizational psychologists and HR practitioners (81, 92). Thus, a positive organization is noteworthy because it serves as a fundamental resource for strengthening workforce wellbeing and meaning in the workplace.

## Limitations and Recommendations for Future Reseach

A few limitations should be considered when interpreting the findings of this research. Firstly, the participants were from different professions and work experiences. Secondly, online interviews can only reveal the understanding of an experience and interpretation of participants' words. It would require alternative methodologies like quantitative and longitudinal design to look more closely at the findings and track changes over times. Thirdly, the research was conducted during a global COVID-19 pandemic that has not been previously experienced by anyone involved and it yielded novel findings that may inform future studies in different circumstances. Lastly, the research was conducted in Malaysia only and on a relatively small sample. Using respondents from different countries and larger samples, may lead to the validation of the proposal that psychological fulfillment should be embedded as part of the Jahoda Latent-deprivation Model, not as an external casual factor.

### Directions for Future Research

Firstly, a cross-country comparative study investigating the effects of a retrenched workforce from different locations and cultural perspectives would extend the body of academic knowledge. Secondly, with the psychological consequences of retrenchment having been evidenced, future study might include psychological intervention underpinning positive psychology theory and approaches. Positive psychology places great importance on human strength and virtues, such as courage, perseverance, hope, and optimism for the future, in maintaining positive mental health as well as promoting psychological resilience (81). Furthermore, cultivating these positive human traits through positive psychology coaching and interventions has been found to enhance wellbeing, as well as reduce stress, anxiety and depressive symptoms (77, 78, 93, 94). Lastly, building on the Latent-deprivation Model, the research suggests adding psychological wellbeing as a new and one of the key latent function and benefits to the revised model under the COVID-19 pandemic. Having established an extended Latent-deprivation model, it is possible to consider positive psychology approaches in developing effective coping strategies to assist in a flourishing professional and personal life.

## Conclusion

This study was conducted to explore the effect of retrenchment due to COVID-19 pandemic on the psychological wellbeing of Malaysians. The study found that both unemployment and the ensuing financial strain that followed retrenchment had a negative effect on the mental health and wellbeing of participants. Besides that, participants also experienced psychological distress due to the economic uncertainty of the COVID-19 pandemic, the change in lifestyle during quarantine, and the fear of being infected with COVID-19 itself. In terms of support, participants reported mental health support, financial support, and career support would be beneficial in improving the psychological wellbeing of the Malaysian workforce. The stigma associated with mental illness is prevalent globally and has led the retrenched workforce to avoid seeking psychological support. The strengths of the present study lay in valuable insights into the experience and wellbeing of the retrenched workforce who had to cope with both unemployment and a global pandemic. The study is a first comprehensive overview of the effect of global pandemic on retrenched workers in Malaysia and as a result makes recommendations on support needed to improve their psychological wellbeing and future integration in the workforce.

## Data Availability Statement

The original contributions presented in the study are included in the article/supplementary material, further inquiries can be directed to the corresponding authors.

## Ethics Statement

The studies involving human participants were reviewed and approved by the Ethics Committee of School of Social Sciences, Heriot-Watt University. The participants provided their written informed consent to participate in this study.

## Author Contributions

KGN and DG conceptualized the study and were actively and equally involved in all stages of the research, including funding application, ethics application, designing of the study, data collection, data analysis, and presentation of results. KGN, DG, SC, and ND equally contributed to manuscript writing, editing, and proofreading. ND also contributed to the data collection and data analysis stage of the research. RK contributed to the interview items design, as well as proofreading in the initial stage of the research. KK, SL, and LWY contributed to the data collection stage of the research. All authors contributed to the article and approved the submitted version.

## Funding

We gratefully acknowledge the funding support by the Global Challenges Research Fund, The Scottish Funding Council, Grant No. SFC: P20GCRF7.

## Conflict of Interest

The authors declare that the research was conducted in the absence of any commercial or financial relationships that could be construed as a potential conflict of interest.

## Publisher's Note

All claims expressed in this article are solely those of the authors and do not necessarily represent those of their affiliated organizations, or those of the publisher, the editors and the reviewers. Any product that may be evaluated in this article, or claim that may be made by its manufacturer, is not guaranteed or endorsed by the publisher.
